# Integrated network analysis identifies hsa-miR-4756-3p as a regulator of FOXM1 in Triple Negative Breast Cancer

**DOI:** 10.1038/s41598-019-50248-3

**Published:** 2019-09-25

**Authors:** Yuanliang Gu, Wenjuan Wang, Xuyao Wang, Hongyi Xie, Xiaojuan Ye, Peng Shu

**Affiliations:** 1 Department of prevention and health care, the People’s Hospital of Beilun District, Beilun Branch Hospital of The First Affiliated Hospital of Medical School Zhejiang University, 1288 Lushan East Road, Beilun District, Ningbo, 315800 China; 2 Molecluar Laboratory, the People’s Hospital of Beilun District, Beilun Branch Hospital of The First Affiliated Hospital of Medical School Zhejiang University, 1288 Lushan East Road, Beilun District, Ningbo, 315800 China; 3 Department of Hematology& Oncology, the People’s Hospital of Beilun District, Beilun Branch Hospital of the First Affiliated Hospital of Medical School of, Zhejiang University, 1288 Lushan East Road, Beilun District, Ningbo, 315800 China

**Keywords:** RNA, Breast cancer

## Abstract

Both aberrantly expressed mRNAs and micro(mi)RNAs play important roles in cancer cell function, which makes integration analysis difficult. In this study, we first applied master regulator analysisalgorithm and confirmed hsa-miR-4756-3p as a candidate miRNA in triple negative breast cancer (TNBC) patients; hsa-miR-4756-3p could regulate TNBC cell line apoptosis, proliferation, migration, and cell cycle as well as suppress TGF-β1 signalling andtumour growth. In TNBC, forkhead box protein M1 (FOXM1)was found to be an hsa-miR-4756-3p target gene, and FOXM1 knockout completely inhibited hsa-miR-4756-3p-induced cell migration and metastasis, TGF-β1 signalling, and epithelial mesenchymal signal activation, which indicated that hsa-miR-4756-3p functions via the FOXM1-TGFβ1-EMT axis.

## Introduction

Breast cancer is a heterogeneous cancer. Based on the molecule subtype technology, breast cancer is divided into 5 subtypes, viz., Luminal A, Luminal B, HER2+, Normal like, and Triple Negative^1^. Among them, triple negative breast cancer(TNBC) is characterized by its poor prognosis^2^, high aggressiveness^3^ after chemotherapies, and insensitivity towards target therapy^4^. Identifying TNBC-specific aberrant expression of genes or micro (mi) RNAs would not only help inthediagnosis of TNBC patients but can also revealnovel druggable targets in TNBC therapy^5^.

miRNAsare a class of non-coding endogenous RNA molecules, whichrangefrom 22 to 25 nucleotidesin length^6^; miRNAsfunction by binding to 3′-UTRs of their target genes and inducing target mRNA degradation^7^. In normal cells, miRNA expression and function are constrained by space and time^8^; they also follow cell- and tissue-specific patterns^9^. However, in tumour cells such as in TNBC, specific miRNAsare critical for tumorigenesis and development^10^. Although many studies have already confirmed that multiple core miRNAs can induce epithelial mesenchymal transition (EMT)^11^, stemness^12^, migration, and invasion^13^, thus, facilitating tumour cell growth and invasion, a more comprehensive study is still needed to investigate the miRNA regulatory network in TNBC.

In this study, we appliedmaster regulator analysis (MMRA) algorithm to identifythe core regulatory miRNAs in TNBC^14^. hsa-miR-4756-3p was confirmed as the top candidate miRNA. We have also elucidatedthe role of hsa-miR-4756-3p in tumour cell function *in vivo*as well as *in vitro*, which is related with TGFβ1 and forkhead box protein M1 (FOXM1).

## Results

### miRNA-mRNA interaction confirmed critical role of hsa-miR-4756-3p in TNBC

**Figure 1:**
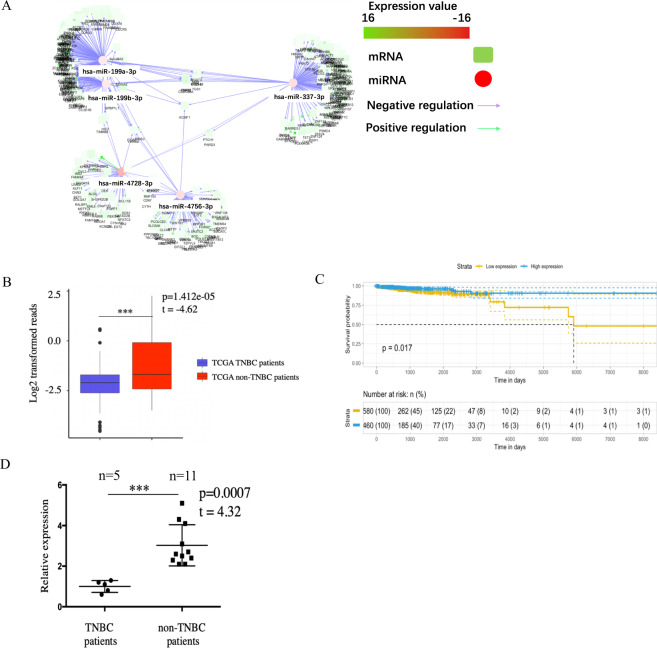
Integrated miRNA-mRNA interaction network confirmed critical role of hsa-miR-4756-3p in TNBC patients. (**A**) Upregulated mRNA and downregulated miRNA were calculated, then the miRNA and mRNA interaction were assessed by microRNA master regulator analysis (MMRA) algorithm. (**B**) hsa-miR-4756-3p expression level from TCGA miRNA matrix was extracted and evaluated by t-test (***p < 0.001). (**C**) hsa-miR-4756-3p expression level and TCGA breast cancer patients’ clinical information was extracted, then hsa-miR-4756-3p relation with patients’ overall survival rate was assessed by univariate cox regression. (**D**) In-house 14 TNBC patients and 3 non-TNBC patient, their primary tumor mRNA was extracted and using QPCR to detect hsa-miR-4756-3p expression level.

To find out the core regulator miRNAs in TNBC, we applied master regulator analysis (MMRA) algorithm. Using this strategy, 5 miRNAs were identified. Except forthat of hsa-miR-4756-3p, the relation of the rest of the miRNAs with breast cancer has already been studied (Fig. 1A). hsa-miR-4756-3p was significantly downregulated in TNBC patients as compared with that in ER+/PR+/HER2+ TCGA breast cancer patients (Fig. 1B), and this low expression of hsa-miR-4756-3p wascorrelated with breast cancer overall survival rate in the TCGA database (Fig. 1C). In our own sample set ofprimary tumoursfrom 5TNBC and 11 non-TNBC patients, hsa-miR-4756-3p expression was 1.00 ± 0.13 and 3.03 ± 0.31, respectively;the expression of hsa-miR-4756-3p was low in TNBC patients (Fig. 1D). This indicated that hsa-miR-4756-3p is a possible tumour suppressor in TNBC.

### Increased expression of hsa-miR-4756-3p induces apoptosis and cell cycle arrest andinhibitscell proliferationand migration *in vitro*

**Figure 2:**
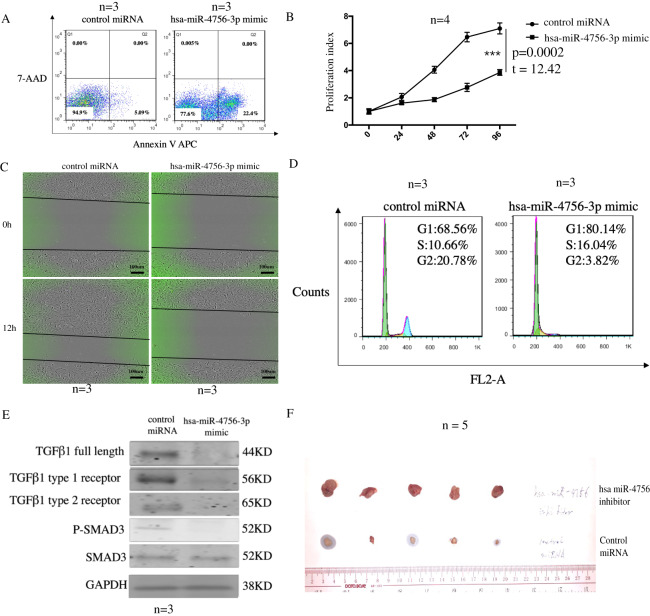
Increased of hsa-miR-4756-3p induced apoptosis, cell cycle arrest and inhibit cell proliferation, migration *in vitro*. Control miRNA and hsa-miR-4756-3p mimic was transfected in TNBC cell line MDA-MB-231 cells for 48 h, then using (**A**)Annexin V APC/7-AAD double staining to detect the change of cell apoptosis. (**B**) CCK8 to detect cell proliferation change. (**C**) Wound healing assay to assess cell migration change. (**D**) High concentration PI staining to assess cell cycle change. (**E**) MDA-MB-231 cell was transfected with hsa-miR-4756-3p mimic, then TGFβ-1 pathway molecules TGFβ-1, TGFβ-1 type 1 receptor, TGFβ-1 type 2 receptor, SMAD3, p-SMAD3 were detected by western blot. (**F**) MDA-MB-231 cell were injected in mammary gland fat pad of nude mice, then control miRNA and hsa-miR-4756-3p inhibitor were inject in nude mice using DOPC liposomes, after 1 months, mice were sacrificed, and primary tumor diameter were assessed.

Expression level of hsa-miR-4756 in TNBC patientshas been found to be low. Further, whether hsa-miR-4756 expression level can affect cell characteristics remains unclear. To address this, we chose the TNBC cell line MDA-MB-231 and transfected it with control miRNA as well ashsa-miR-4756-3p mimic for 48 h. First, we confirmed the cell apoptosis rate in control miRNA andhsa-miR-4756-3p groups, which were 5.03 ± 0.18 and 23.47 ± 1.46, respectively;cell apoptosis was increased in the case of transfection with hsa-miR-4756-3p (Fig. 2A). Cell proliferation in the hsa-miR-4756-3p mimic group was suppressed (Fig. 2B). Forassessing cell migration, we used wound healing assay. After culture in 37 °C for 12 h, migration velocity in the control and hsa-miR-4756-3p mimic groups was found to be 4.81 ± 0.17 and 1.19 ± 0.09, respectively; cell migration in the hsa-miR-4756-3p group was suppressed (Fig. 2C). Regarding the cell cycle, the proportion of cells in the G2 phasein the control and hsa-miR-4756-3p mimic group was found to be 18.33 ± 1.13 and 4.35 ± 0.39, respectively (Fig. 2D). These results indicated that the increased hsa-miR-4756-3p expression can trigger inhibition of cell proliferation and migration and induction of cell apoptosis as well as cell cycle progression. As hsa-miR-4756-3p widely takes part in cell functions such as invasion, migration and proliferation, and TGFβ-1 signal is an important pathway in tumour development^8^. First, we detected the changes in the TGFβ-1 pathway in control and hsa-miR-4756-3p mimic groups;the protein levels of TGFβ-1 full length, TGFβ-1 type1 receptor, and TGFβ-1 type 2 receptor were decreased as was the related SMAD3 phosphorylation, which indicated down-regulation ofthe TGFβ-1 pathway after increase in hsa-miR-4756-3p expression (Fig. 2E), all of these western blot were repeated by 3 times. We then injected MDA-MB-231 cells into themammary glandsof nude mice, nude mice were treated withcontrol miRNAand hsa-miR-4756-3p inhibitor. After 1 month, the tumour sizes in the hsa-miR-4756-3p group were found to be significantly smaller (Fig. 2F).

### FOXM1 serves as an hsa-miR-4756-3p target gene in TNBC

**Figure 3:**
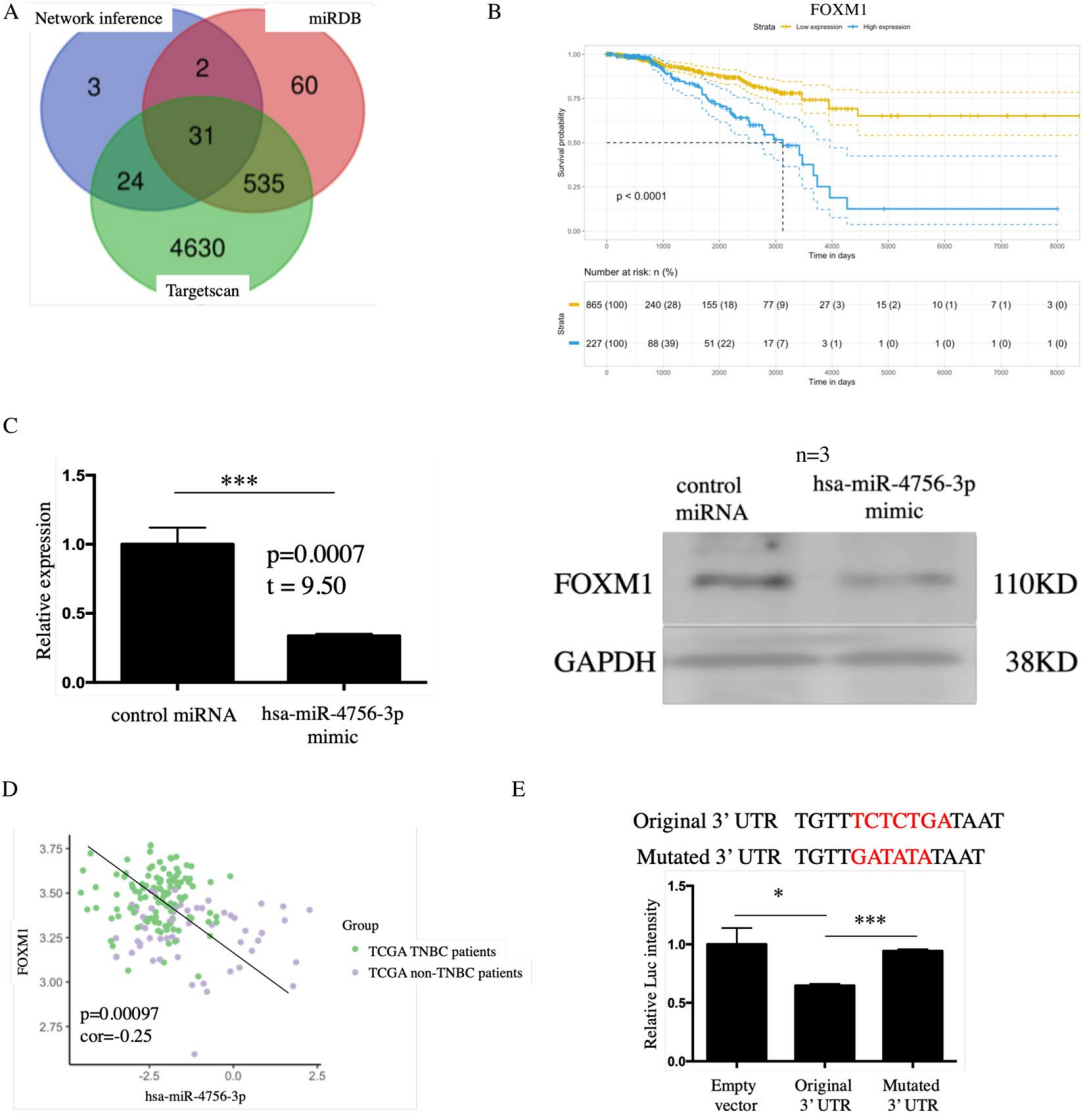
FOXM1 severed as hsa-miR-4756-3p target gene in TNBC patients. (**A**) 60 network inference mRNA, 5220 targetscan database predict mRNA and 628 miRDB predict mRNA were combine together and 31 mRNA overlapped, all of them were assessed relation with TCGA breast cancer patients’ overall survival rate using univariate cox regression. (**B**) FOXM1 was the only one which related with patient’s overall survival rate. (**C**) MDA-MB-231 cell were transfected with hsa-miR-4756-3p mimic, then expression of FOXM1 in mRNA and protein level were detected by QPCR and western blot. (**D**) Expression data of hsa-miR-4756-3p and FOXM1 in TCGA breast cancer patient were extracted, and correlation was assessed by pearson correlation analysis. (**E**) original and mutation hsa-miR-4756-3p binding site in FOXM1 3′-UTR were cloned to luciferase vector (up), and luciferase intensity was assessed by microplate reader (down).

Further, to identify hsa-miR-4756-3p target genes in TNBC, we first extractedpossible hsa-miR-4756-3p target genes, which contained 60 network inference mRNAs, 5220 TargetScan mRNAs, and 628 miRDB mRNAs. After combining them, we found 31 overlapping mRNAs (Fig. 3A). Among these 31 genes, only the expression of FOXM1 correlated with TCGA breast cancer patients’ overall survival rate (Fig. 3B). Subsequently, we detected FOXM1 expression change after an increment in miR-4756-3p expression. As for mRNA levels, the values detected for controland hsa-miR-4756-3p groups were 1.00 ± 0.07 vs 0.34 ± 0.01; in protein levels, after repeated experiments for 3 times and calculated gray-value, compared with control miRNA, hsa-miR-4756-3p mimic also suppressed FOXM1 expression in protein level (control vs mimic = 1.00 ± 0.17 vs 0.54 ± 0.21, p value = 0.042)(Fig. 3C). In the TCGA database, we selected TNBC (ER−, PR−, HER2−) and non-TNBC (ER+, PR+, HER2+)patients, and extract their mRNA expression value, FOXM1 and hsa-miR-4756-3p is negatively correlated, and most of the FOXM1 high/hsa-miR-4756-3p low samples are concentrated in TNBC patients (Fig. 3D). To prove the direct interaction between hsa-miR-4756-3p and FOXM1, we mutated the FOXM1 3′-UTR (Fig. 3E up). The original FOXM1 3′-UTR andmutated FOXM1 3′-UTR were then transfected into MDA-MB-231 cells. As compared with the empty vector control, treatment with the wild type hsa-miR-4756-3p significantly inhibited luc intensity; however, the variant failed to inhibit the luc intensity (Fig. 3E down), which indicated direct interaction between FOXM1 and hsa-miR-4756-3p.

### hsa-miR-4756-3p regulates TNBC metastasis *in vitro* and *in vivo* through the FOXM1-TGFβ1-Smad3-EMT pathway

**Figure 4:**
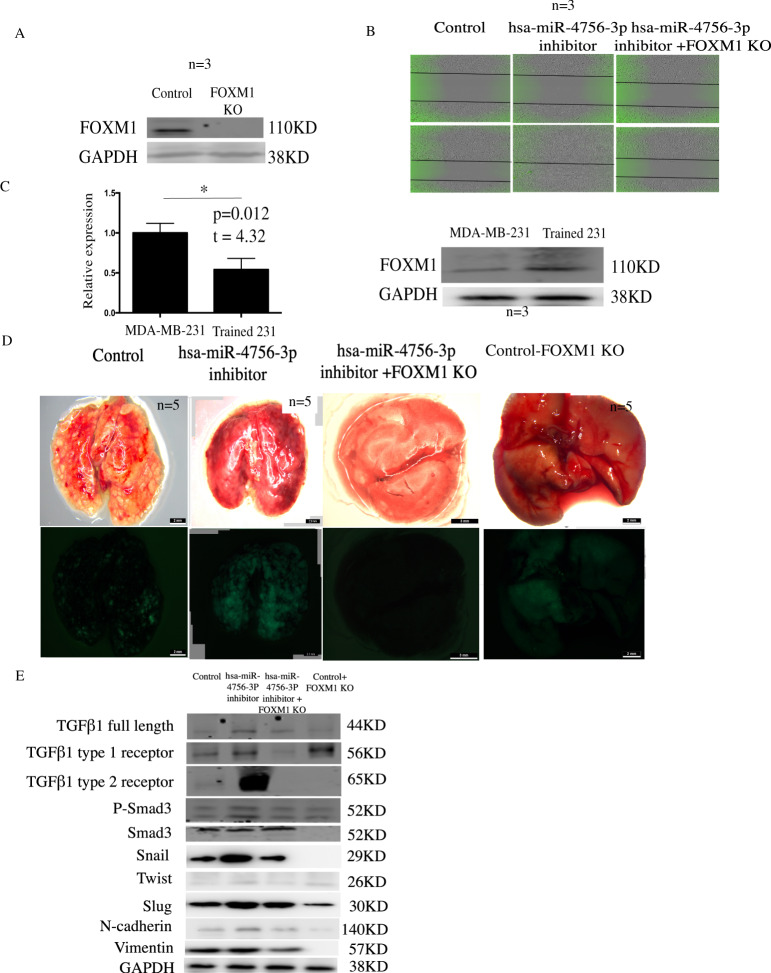
hsa-miR-4756-3p regulated TNBC metastasis *in vitro* and *in vivo* through FOXM1-TGFβ1-Smad3-EMT pathway. (**A**) Control sgRNA and FOMX1 KO were transfected in MDA-MB-231 cells, after single clone selection, using western blot to detect change of FOXM1 expression. (**B**) MDA-MB-231 cells was divided into control, hsa-miR-4756-3p inhibitor, hsa-miR-4756-3p inhibitor + FOXM1 KO group, control group transfected with control miRNA, hsa-miR-4756-3p inhibitor transfected with hsa-miR-4756-3p inhibitor, hsa-miR-4756-3p inhibitor + FOXM1 KO group was 231-FOXM1 KO transfected with hsa-miR-4756-3p inhibitor, then using wound healing assay to assess the migration change. (**C**) Employing QPCR and western blot to find the hsa-miR-4756-3p (left) and FOXM1(right) expression in 231 and trained 231 cells. (**D**) 15 nude mice were divided into control, hsa-miR-4756-3p inhibitor, hsa-miR-4756-3p inhibitor + FOXM1 KO group, control, hsa-miR-4756-3p inhibitor group were injected trained 231 in mammary gland fat pad, hsa-miR-4756-3p inhibitor + FOXM1 KO group were injected with FOXM1 KO trained 231 cells, then control miRNA was injected control nude mice using DOPC liposomes, hsa-miR-4756-3p inhibitor was injected in other 2 groups, after 2 months, mice lung metastasis were detected. (**E**) In control, hsa-miR-4756-3p inhibitor, hsa-miR-4756-3p inhibitor + FOXM1 KO group nude mice primary tumor, protein was extracted and TGFβ1 signal pathway, EMT pathway were assess.

After we confirmed that FOMX1 is a direct target of hsa-miR-4756-3p, we created FOMX1 KO MDA-MB-231 cells (Fig. 4A). Using wound healing assay, we confirmed that inhibition of hsa-miR-4756-3p could promote cell migration, which corresponded with previous results. However, KO of FOXM1 completely suppressed the cell migration (Fig. 4B). Then, we constructed highly metastasis trained MDA-MB-231 cell as described in the methods section. By using qPCRand western blotting, hsa-miR-4756-3pexpression was found to be lower and FOXM1 was found to be highly expressedin trained 231 cells (Fig. 4C). Trained 231 cells were injected in nude mice with or without FOXM1 KO, and these mice were treated with or without hsa-miR-4756-3p inhibitor. After 2 months, lung metastasis in hsa-miR-4756-3p inhibitor-treated mice wassignificantly increased, but KO of FOXM1 completely eliminated the lung metastasis (Fig. 4D), indicating that the function of hsa-miR-4756-3p is mediated by FOXM1. We also checked TGFβ1 signal and EMT signal in primary tumours from control, hsa-miR-4756-3p inhibitor, and hsa-miR-4756-3p inhibitor + FOXM1 KO mice. After the suppression of hsa-miR-4756-3p, TGFβ1 signalsand EMT signalswere found to beincreased. FOXM1 KO inhibited both TGFβ1 signalsand EMT signals (Fig. 4E).

## Discussion

Compared withthat of other subtypes of breast cancer, prognosis of TNBC is poorer, and therefore, the discovery of new druggable targets is urgent. Here, we applied MMRA algorithm and found5 core regulatory miRNAs, viz., hsa-miR-199a-3p, hsa-miR-199b-3p, hsa-miR-377-3p, hsa-miR-4728-3p, and hsa-miR-4756-3p in TNBC. Among these 5 miRNAs, hsa-miR-4756-3p is a relatively newly identified miRNA; its function and relation with TNBC has not been studied yet. Based on hsa-miR-4756-3p expression in TCGA TNBC patients and our in-house patients, hsa-miR-4756-3p was found to be down-regulated in TNBC primary tumour. The down-regulation of hsa-miR-4756-3p also corelated with the patients’ overall survival rate, which indicates that hsa-miR-4756-3p might act as a tumour suppressor.

To further clarify the role of hsa-miR-4756-3p in TNBC, we designed hsa-miR-4756-3p mimic and transfected it into in MDA-MB-231 cells. The overexpression of hsa-miR-4756-3p could induce 231 cell apoptosis and cell cycle arrest and suppress cell migration and proliferation. These results indicated that hsa-miR-4756-3p is widely involved in TNBC cell function*in vitro*. Hence, we assumedthat hsa-miR-4756-3p function functions through an oncogenic pathway. Upon overexpression ofhsa-miR-4756-3p, TGF-β1, and TGF-β1 type 1 and 2 receptors were down-regulated. The TGF-β1 downstream signal, SMAD3 phosphorylation, also decreased, indicating the effects ofhsa-miR-4756-3p on the TGF-β1 pathway. After combination of network inference mRNA and target gene deduction for hsa-miR-4756-3p using database, 31 overlapping mRNAs were found;only FOXM1 correlated with the patients’ survival. Through luc mutation assay, we confirmed direct interaction between hsa-miR-4756-3p and FOXM1.

FOXM1 is a well-studied gene in TNBC. It is known to be highly expressed primarily in TNBC as compared with that in other subtypes. After treatment with FOXM1 inhibitor thiostrepton, the EMT markers ZEB1 andvimentin, and cell cycle markers CDK1 and PLK1 were found to bedecreased^15^. The underlying mechanism of action of FOXM1 in breast cancer involves sustained activation of SMAD3/SMAD4 activity, which further induces TGF-β-dependent EMT and promotes metastasis^16^. On the other hand, overexpressed FOXM1 can recruit tumour associated macrophages (TAM), which can also secrete TGF-β1 and promote tumour cell invasion^17^. Using FOXM1 KO 231 cells, we found that cell migration induced by hsa-miR-4756-3p was mediated byFOXM1. Besides, FOXM1 KO eliminated metastatic cells from trained 231-injected nude mice, which further indicated the importance of FOXM1 in hsa-miR-4756-3p function. By western blotting, we also showedthat the levels of the TGF-β1 signalsincluding TGF-β1, TGF-β1 type1 and 2 receptors, the downstream Smad3 pathway, the EMT pathway, which included Snail, Twist, Slug, N-cadherin, and vimentin, were all increased in hsa-miR-4756-3p inhibitor-treated mice.

Based on these results, we proved the lower expression of hsa-miR-4756-3p in TNBC patients’ primary tumours. We also confirmed that hsa-miR-4756-3p could regulate TNBC cell line function via the FOXM1-TGFβ1-EMT axis.

## Methods

### Ethics statement

All the breast cancer patients included in this study signed informed consent forms. The present experiments including human and animal subjects were approved by the Ethics Committee of Ningbo Beilun People’s Hospital. All the following protocols were approved in advance by the Ningbo Beilun People’s Hospital, Ningbo city, Zhejiang province, China. And all the methods in this study were performed in accordance with the relevant guidelines and regulations formulated by the Ningbo Beilun People’s Hospital, Ningbo city, Zhejiang province, China.

### miRNA-mRNA interaction network analysis

All the bioinformatics analysis procedureswere carried out using R 3.4.3 software. To find out core regulatory miRNAs in TNBC, we applied miRNA-mRNA interaction network analysis (1) TCGA (The Cancer Genome Atlas) level-3 miRNA and mRNA expression value, patients clinical information were downloaded by GDCRNATools package (https://github.com/Jialab-UCR/GDCRNATools)^18^. (2)After normalization, TNBC (ER−/PR−/HER2−) and non-TNBC (ER+/PR+/HER2+) patients’ information was extracted, differential expression of miRNAs and mRNAs was calculated using limma package.(3) FC > 1 and p < 0.05 gene were subscribed to MMRA^14^. Using stepwise linear regression analysis, candidate miRNAs and mRNAs were visualized by MiRComb package. By searching previous literature, 5 miRNAs were confirmed;among them, hsa-miR-4756-3p had not been studied previously in TNBC. (4) After confirming the candidate miRNA, we extracted possible target genes from TargetScan (http://www.targetscan.org/), miRDB (http://mirdb.org/), and mRNA from network inference (highly correlated with miRNA). (5) Overlap of mRNA was visualized by Venn diagram (http://bioinformatics.psb.ugent.be/webtools/Venn/). Correlation of miRNA and top candidate mRNA were calculated by Pearson correlation and visualized by ggplot2 package. (6) miRNA and target mRNA relation with breast cancer patients’ overall survival rate was calculated by survival package and visualized by survminer package.

### Patient samples

5 cases of TNBC (ER−/PR−/HER2−) and 11 cases of non-TNBC (ER+/PR+/HER2+) patients’ primary were obtained from Ningbo Beilun People’s Hospital. All the selected patients were females; patients’ average age in TNBC and non-TNBC groups was 65.17 ± 13.51 and 63.81 ± 12.43, respectively.

### Cell line and miRNA transfection

Human TNBC cell line MDA-MB-231 was cultured in DMEM (Invitrogen, Carlsbad, CA, USA) containing 10%FBS (HyClone Laboratories, Logan, UT, USA). MDA-MB-231 cells were divided into control miRNA and hsa-miR-4756-3p mimic groups, and transfected with 100 nM control miRNA and hsa-miR-4756-3p mimic (Sigma-Aldrich, St Louis, MO, USA) using Lipofectamine 2000(Invitrogen, Carlsbad, CA, USA), and hsa-miR-4756-3p inhibitor was also purchased from Sigma-Aldrich company, transfection procedure was according to hsa-miR-4756-3p mimic transfection.

### q-RT-PCR

Total RNA from patients’ primary tumour and cell line was extracted using Trizol Reagent (Invitrogen, Carlsbad, CA, USA), after mRNA extraction. (1)For the miRNA expression detection, Taqman miRNA assays kit (Life Technologies, Carlsbad, CA, USA) was used according to user protocol; RNU6B served as housekeeping gene. (2) For the mRNA expression, mRNA was reverse transcribed using Transcriptor First Strand cDNA Synthesis Kit (Roche Life Science, Indianapolis, Indiana, USA). Then the qPCR was accomplished with ABI 7500 fast system using SYBR-Green dye (Roche Life Science, Indianapolis, Indiana, USA). Finally mRNA expression value was calculated by 2^−ΔΔCT^ method. The primer sequencesused for FOXM1 were F-5′-TCTGCCAATGGCAAGGTCTCCT-3′, R-5′-CTGGATTCGGTCGTTTCTGCTG-3′ and that for housekeeping gene GAPDH were F-5′-GTCTCCTCTGACTTCAACAGCG-3′, R-5′-ACCACCCTGTTGCTGTAGCCAA-3′. All the experiments were repeated 3 times and select median as criteria.

### Western blotting

In the western blotting, (1) cells were lysed by RIPA buffer and loading buffer mix, proteins were separated using 12% SDS-PAGE electrophoresis, and the bands were transferred to Polyvinylidene difluoride (PVDF) membrane. (2) Membrane was blocked by 5% BSA for 2 h, andlabelled with primary antibodies. Antibodies against TGFβ-1(ab92486), TGFβ-1 type 1 receptor (ab31013), TGFβ-1 type 2 receptor (ab78419) were purchased from Abcam (Cambridge, Milton, UK), whereas those for Snail (C15D3), Twist (46702), Slug (C19G7), N-cadherin (D4R1H), Vimentin (D21H3), SMAD3(9513), p-SMAD3(9520), GAPDH (2118) were purchased from Cell SignalingTechnology (MA, USA). After overnight incubation with primary antibodies at 4 °C, membrane was subsequently incubated with secondary antibody; anti-rabbit or anti-mouse antibodies were all purchased from ZhongShan Golden Bridge Biotechnology (Beijing, China), and finally gene expression value in protein level was quantified as gray-value by using Image J software. All the experiments were repeated 3 times and select median as criteria, and full length of western blot figure was represented in Supplementary Information.

### Cell apoptosis assay, proliferation assay, and wound healing assay

(1) In the apoptosis assay, control and hsa-miR-4756-3p mimic group cells were transfected with control miRNA and hsa-miR-4756-3p mimic for 48 h, washed with PBS solution 2 times, and subsequently labelled with APC-conjugated Annexin-V (BD Biosciences, San Jose, CA)/propidiumiodide(PI). Then cells were detected using FACS Calibur (BD Biosciences, San Jose, CA);apoptotic cells were quantified by APC + cell ratio. (2) In the cell proliferation assay, after transfection of miRNA, cells were dispersed in 96-wells plate. Each well had 1500 cells, and totally we set 0, 24, 48, 72, 96 h time point. When reach the time point, 10% volume of Cell Counting Kit-8 (CCK-8)(Dojindo molecular technologies, Rockville, MD, USA) was added to it and the cells were cultured for 2 h at 37 °C. The cell proliferation was then detected by Microplate Reader for OD at 450 nm; cell proliferation index was quantified as (reads from time points)/(reads from 0 h). (3) In the wound healing assay, after control miRNA and hsa-miR-4756-3p mimic cells attached to the plate, a scratch was made on cell monolayer and migration velocity was quantified as (cell movement distance in 12 h quantified by scale on the picture)/hours. All the experiments were repeated 3 times and select median as criteria.

### Cell cycle assay

For the cell cycle assay, cells were harvested and resuspended in 75% ethanol + 5%FBS, and then incubated at −20 °C for overnight. The cells were then stained with high concentration PI (Sigma-Aldrich, St Louis, MO, USA). The cell cycle was detected by FACS caliber and analysed by FlowJo 7.6.1 software. All the experiments were repeated 3 times and select median as criteria.

### Luciferase reporter assay

In the luciferase report assay (1) Cells were transfected with control miRNA and hsa-miR-4756-3p mimic. At the same time, cells were transfected with empty vector, original FOXM1 3′-UTR, and mutation type of FOXM1 3′-UTR (Switchgear Genomics, Menlo Park, CA, USA); all the cells were also transfected with control vector. (2) After 48 h, cells were lysed and luciferase intensity was detected by LightSwitch Dual Luciferase assay kit (Biotek, Winooski, VT, USA) using the microplate reader. (3) Luciferase intensities were first normalized by Cypridina TK control, and then by using empty vector sample reads as control. Finally the changesin the Luc intensity were quantified as (sample normalized reads)/(empty vector normalized reads). All the experiments were repeated 3 times and select median as criteria.

### Nude mice experiment

In the mice experiment, all the experiment complied with National Institutes of Health guide for the care and use of Laboratory animals (NIH Publications No. 8023, revised 1978). Female nude mice (Beijing Wei-tong Li-hua Laboratory Animals and Technology, Beijing, China) were first injected with 0.5 million MDA-MB-231 cell in the mammary gland fat pad; the mice were divided into control miRNA and hsa-miR-4756-3p mimic groups, each group had 5 mice. The mice were then treated with control miRNA and hsa-miR-4756-3p inhibitor intraperitoneally using DOPC liposomes for 2 times a week. After 1 month, mice were dissected and primary tumour diameters were calculated. The immunohistochemistry (IHC) experiment was carried out by Xue bang company (Beijing, China).

### Construction of highly metastatic MDA-MB-231 cells

Normally metastasis node of MDA-MB-231 cell is diffused and unstable. To get highly metastasis ability 231, we first injected labelled MDA-MB-231 cell with GFP, and then injected it in the mammary gland of nude mice. After 2 months, we extracted GFP + cells from lung using FACS sorting (Aria 3, BD Biosciences, San Jose, CA). We then injected the selected cells in nude mice mammary gland again. After 3 such rounds, we got 231 with high metastasis ability; we termed these cells as trained 231 cells.

### FOMX1 KO cell line construction

To get FOXM1 KO cell lines, 3 sgRNA sequences were selected: sg1: GTCAAGTAGCGATTGGCACT, sg2: CTACAGGTTGAGGAGCCCTC, sg3: GGGCCCCTGCAGCGTTAAGC. All 3 sgRNAs oligos were cloned in to lentiCRISPR v2 vector (Addgene, 52961) following manufacturer’s user instructions. Then single clone selection was applied, and positive clones were selected using western blot. Finally sg2 based clone was selected in 231 and trained 231 cells.

### Statistical analysis

All the statistical analyses were carried out using R 3.4.3. Significance levels were determined by Student’s t test and ANOVA analysis, *p < 0.05, **p < 0.01, ***p < 0.001.

## Supplementary information

Integrated network analysis identifies hsa-miR-4756-3p as a regulator of FOXM1 in Triple Negative Breast Cancer
