# Author Correction: Integrated network analysis identifies hsa-miR-4756-3p as a regulator of FOXM1 in Triple Negative Breast Cancer

**DOI:** 10.1038/s41598-021-99328-3

**Published:** 2021-10-14

**Authors:** Yuanliang Gu, Wenjuan Wang, Xuyao Wang, Hongyi Xie, Xiaojuan Ye, Peng Shu

**Affiliations:** 1 Department of Prevention and Health Care, the People’s Hospital of Beilun District, Beilun Branch Hospital of the First Affiliated Hospital of Medical School Zhejiang University, 1288 Lushan East Road, Beilun District, Ningbo, 315800 China; 2 Molecluar Laboratory, the People’s Hospital of Beilun District, Beilun Branch Hospital of the First Affiliated Hospital of Medical School Zhejiang University, 1288 Lushan East Road, Beilun District, Ningbo, 315800 China; 3 Department of Hematology and Oncology, the People’s Hospital of Beilun District, Beilun Branch Hospital of the First Affiliated Hospital of Medical School Zhejiang University, 1288 Lushan East Road, Beilun District, Ningbo, 315800 China

Correction to: *Scientific Reports* 10.1038/s41598-019-50248-3, published online 25 September 2019

The original version of this Article contained an error in Figure 2F where the cell line for trained MDA-MB-231 was used instead of MDA-MB-231. The original Figure 2 and accompanying legend appear below.

Similarly, in Figure 4D, the MDA-MB-231 cell line was used instead of the trained MDA-MB-231. The original Figure 4 and accompanying legend appear below.

The original Article has been corrected.

**Figure 2:**
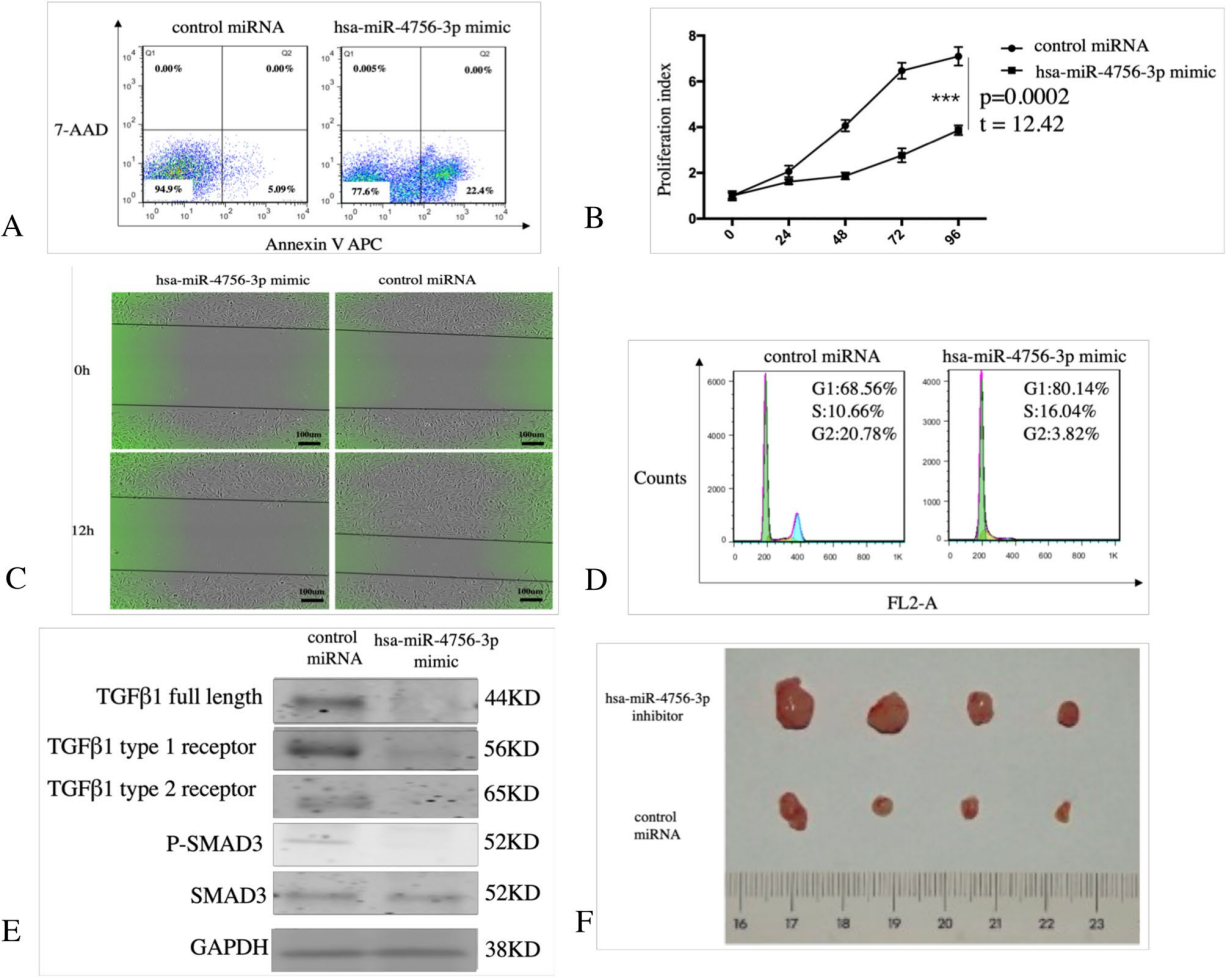
Increased of hsa-miR-4756-3p induced apoptosis, cell cycle arrest and inhibit cell proliferation, migration *in vitro*. Control miRNA and hsa-miR-4756-3p mimic was transfected in TNBC cell line MDA-MB-231 cells for 48 h, then using (**A**) Annexin V APC/7-AAD double staining to detect the change of cell apoptosis. (**B**) CCK8 to detect cell proliferation change. (**C**) Wound healing assay to assess cell migration change. (**D**) High concentration PI staining to assess cell cycle change. (**E**) MDA-MB-231 cell was transfected with hsa-miR-4756-3p mimic, then TGFβ-1 pathway molecules TGFβ-1, TGFβ-1 type 1 receptor, TGFβ-1 type 2 receptor, SMAD3, p-SMAD3 were detected by western blot. (**F**) MDA-MB-231 cell were injected in mammary gland fat pad of nude mice, then control miRNA and hsa-miR-4756-3p inhibitor were inject in nude mice using DOPC liposomes, after 1 months, mice were sacrificed, and primary tumor diameter were assessed.

**Figure 4:**
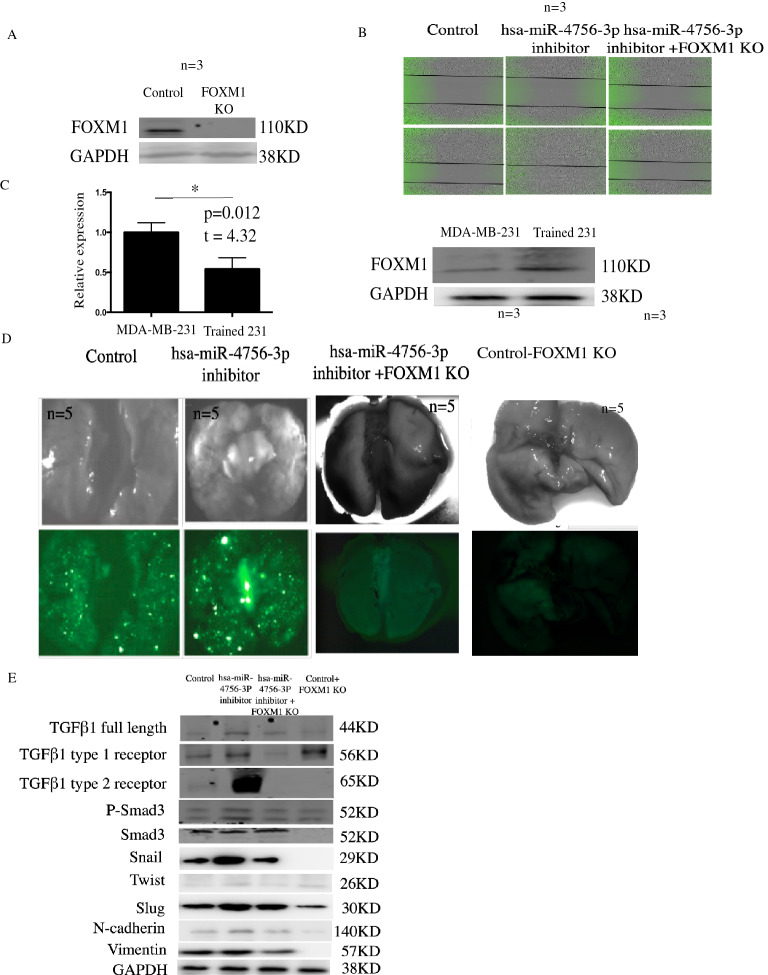
hsa-miR-4756-3p regulated TNBC metastasis *in vitro* and *in vivo* through FOXM1-TGFβ1-Smad3-EMT pathway. (**A**) Control sgRNA and FOMX1 KO were transfected in MDA-MB-231 cells, after single clone selection, using western blot to detect change of FOXM1 expression. (**B**) MDA-MB-231 cells was divided into control, hsa-miR-4756-3p inhibitor, hsa-miR-4756-3p inhibitor + FOXM1 KO group, control group transfected with control miRNA, hsa-miR-4756-3p inhibitor transfected with hsa-miR-4756-3p inhibitor, hsa-miR-4756-3p inhibitor + FOXM1 KO group was 231-FOXM1 KO transfected with hsa-miR-4756-3p inhibitor, then using wound healing assay to assess the migration change. (**C**) Employing QPCR and western blot to find the hsa-miR-4756-3p (left) and FOXM1(right) expression in 231 and trained 231 cells. (**D**) 15 nude mice were divided into control, hsa-miR-4756-3p inhibitor, hsa-miR-4756-3p inhibitor + FOXM1 KO group, control, hsa-miR-4756-3p inhibitor group were injected trained 231 in mammary gland fat pad, hsa-miR-4756-3p inhibitor + FOXM1 KO group were injected with FOXM1 KO trained 231 cells, then control miRNA was injected control nude mice using DOPC liposomes, hsa-miR-4756-3p inhibitor was injected in other 2 groups, after 2 months, mice lung metastasis were detected. (**E**) In control, hsa-miR-4756-3p inhibitor, hsa-miR-4756-3p inhibitor + FOXM1 KO group nude mice primary tumor, protein was extracted and TGFβ1 signal pathway, EMT pathway were assess.

