# Supplementary material for: Integrated network analysis identifies hsa-miR-4756-3p as a regulator of FOXM1 in Triple Negative Breast Cancer
Source: Sci Rep. 2019 Sep 25;9:13830. doi: 10.1038/s41598-019-50248-3 (PMC6761188; doi:10.1038/s41598-019-50248-3)
Supplement: Supplementary file 1 — Integrated network analysis identifies hsa-miR-4756-3p as a regulator of FOXM1 in Triple Negative Breast Cancer [file 41598_2019_50248_MOESM1_ESM.pdf]

Integrated network analysis identifies hsa-miR-4756-3p as a regulator of FOXM1 in Triple Negative Breast Cancer

Yuanliang Gu1, Wenjuan Wang2, Xuyao Wang2, Hongyi Xie2, Xiaojuan Ye3, Peng Shu2

1 Department of prevention and health care, the People’s Hospital of Beilun District, Beilun Branch Hospital of The First Affiliated Hospital of Medical School Zhejiang University,1288 Lushan East Road, Beilun District, Ningbo 315800, China

2 Clinical Laboratory, the People’s Hospital of Beilun District, Beilun Branch Hospital of The First Affiliated Hospital of Medical School Zhejiang University, 1288 Lushan East Road, Beilun District, Ningbo 315800, China

3 Department of Hematology & Oncology, the People's Hospital of Beilun District, Beilun Branch Hospital of the First Affiliated Hospital of Medical School of ,Zhejiang University, 1288 Lushan East Road, Beilun District, Ningbo 315800, China

*Address correspondence to:

Peng Shu, MD.

Clinical Laboratory, the People’s Hospital of Beilun District, Beilun Branch Hospital of The First Affiliated Hospital of Medical School Zhejiang University, 1288 Lushan East Road, Beilun District, Ningbo 315800, China

E-mail: m17757498873@163.com

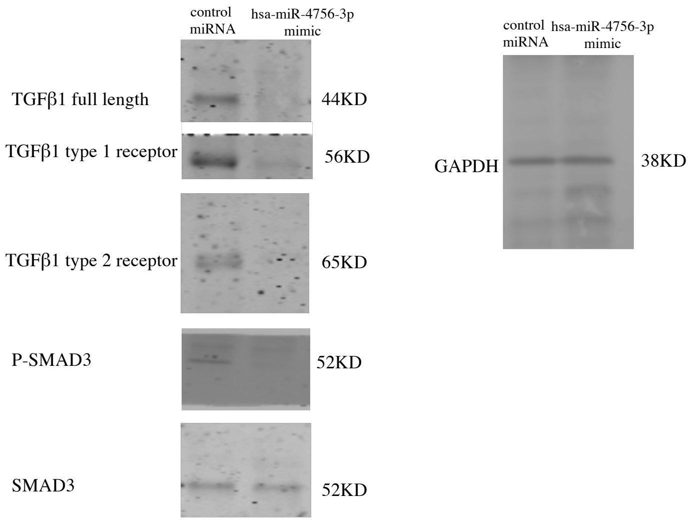

Fig S1 Full length western blot of Figure 2E

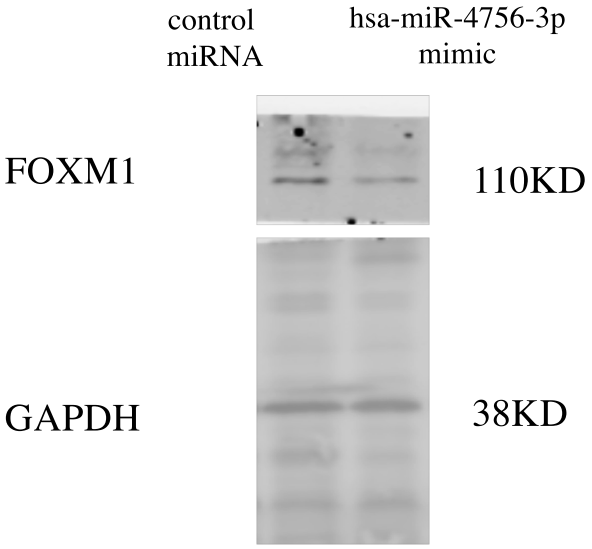

Fig S2 Full length western blot of Figure 3C

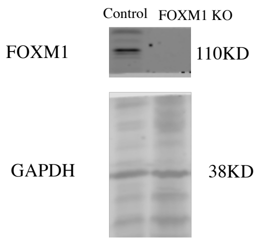

Fig S3 Full length western blot of Figure 4A

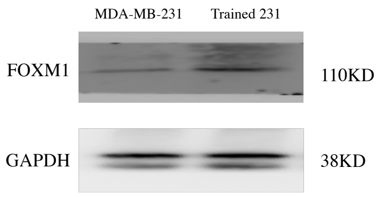

Fig S4 Full length western blot of Figure 4C

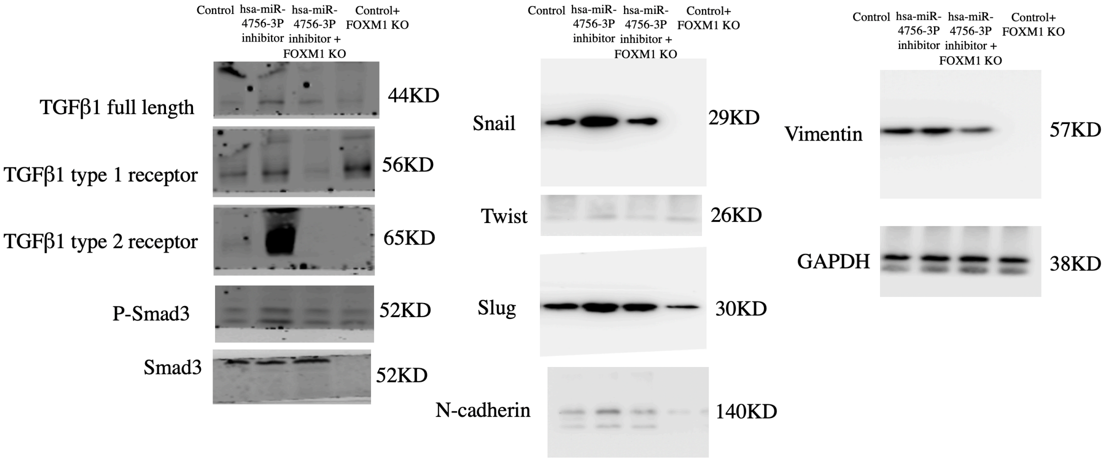

Fig S5 Full length western blot of Figure 4E
